# Acute Appendicitis in a Double Appendix: A Case Report

**DOI:** 10.7759/cureus.49799

**Published:** 2023-12-01

**Authors:** Jolan S Alsaud, Haya Alnumayr, Shatha Aljamaan, Mayar Aloufi, Abduallah Momtaz

**Affiliations:** 1 College of Medicine, Qassim University, Qassim, SAU; 2 General Surgery, Ayoun Al Jawa General Hospital, Qassim, SAU

**Keywords:** cave-wallbridge classification, two appendicitis, diagnosis of acute appendicitis, abdominal pain, appendectomy, acute appendicitis, inflammation, double appendixes

## Abstract

Acute appendicitis is one of the most common non-traumatic emergency surgical pathologies, and appendix duplication is a congenital defect that is challenging to diagnose. It is often discovered incidentally during laparoscopy or laparotomy. If a double appendix or other associated anomalies are not detected, complications can be severe and potentially fatal. There are few cases of appendicular duplication. We report the incidental discovery of a double appendix in a 36-year-old man who came to the emergency department complaining of sharp right iliac fossa pain for three days and other features of appendicitis. During surgery, it was surprisingly discovered that he had two appendices. Both were inflamed, and an appendectomy was done for both of them. This case emphasizes the significance of this condition as a misdiagnosis might result in serious, potentially fatal consequences for the patient in addition to other health and legal issues.

## Introduction

The vermiform appendix forms as a conical extension from the caecal diverticulum's apex, which originates from the antimesenteric border of the proximal part of the post-arterial segment of the midgut. A recent assessment found that between 0.004% and 0.009% of the appendices were duplicated. Diagnosing a double appendix is complicated and, most of the time, discovered during surgery, which makes the complications that arise from an unknown double appendix more severe and life-threatening for the patient [1-5]. This case report presents a case of inflamed appendix duplication defects. Additionally, it highlights the importance of this condition because a misdiagnosis can have serious, even life-threatening, effects on the patient, not to mention medical and legal problems.

## Case presentation

A 36-year-old man presented to the emergency department complaining of sharp right iliac fossa pain for three days. The pain started in the periumbilical region and then migrated to the right iliac fossa, with nausea, anorexia, and vomiting, without urinary symptoms or altered bowel habits. He was not known to have any chronic illnesses or had any previous surgery. On examination, there was elevated temperature and tenderness in the right iliac fossa with positive rebound tenderness, which indicated peritoneum irritation. Laboratory investigations showed leukocytosis and mild anemia with normal liver enzymes, electrolytes, and coagulation profiles. This patient's Alvarado score [6] was 9 (Table 1).

**Table 1:** Alvarado Score Reference [6]

Feature	Score
Symptoms	
Migration of pain	1
Anorexia	1
Nausea	1
Signs	
Tenderness on the right lower quadrant	2
Rebound pain	1
Elevated temperature	1
Laboratory values	
Leukocytosis	2
Shift of white blood cell count to the left	1
Total	10

**Figure 1:**
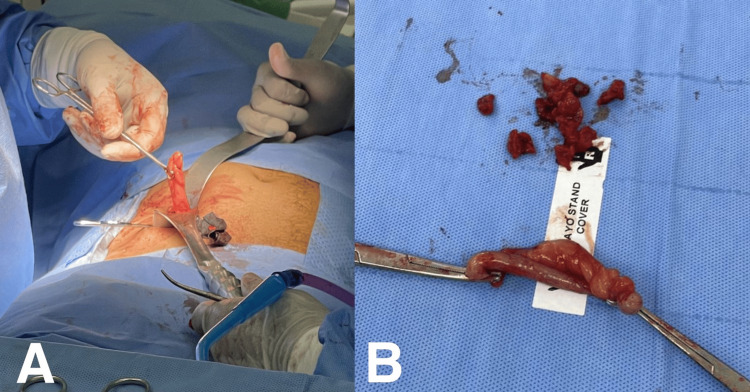
Intraoperative appendectomy pictures A: Intraoperative picture showing the appendices; B: The gross specimens of the two appendices and the fecalith. Image credit: Abduallah Momtaz

**Figure 2:**
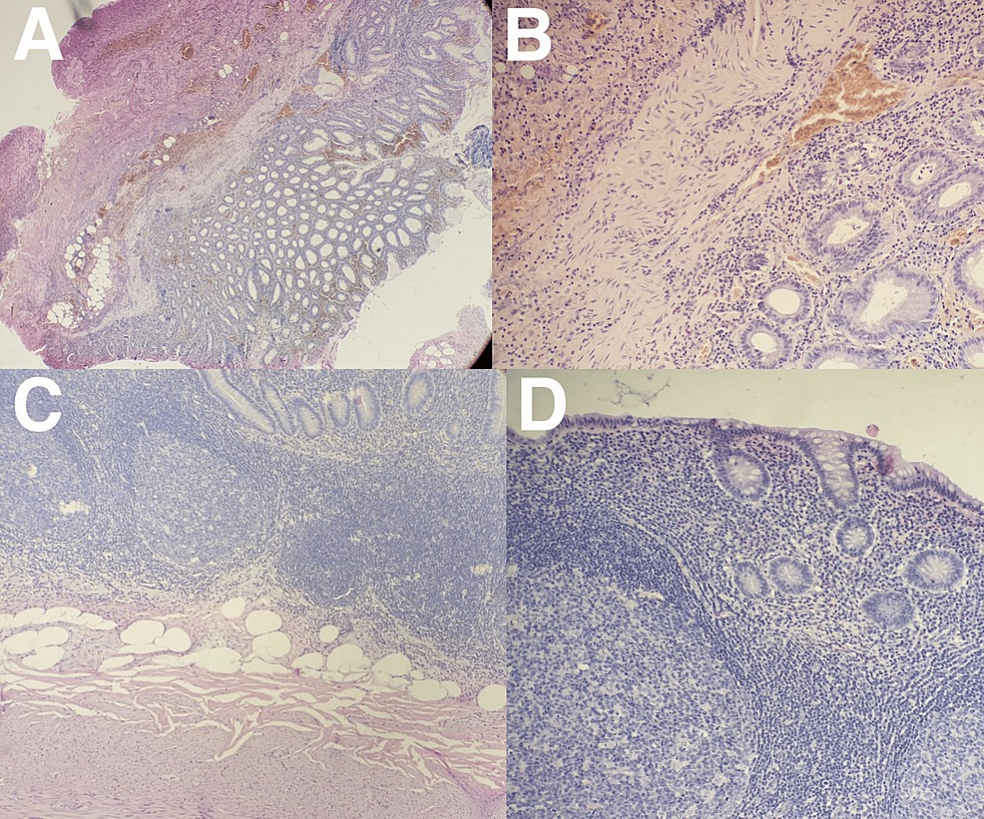
Histopathological examination A: Low-power microscopic view of the first specimen; B: High-power microscopic view of the first specimen showing the structure of appendix and acute inflammatory infiltrate: C: Low-power microscopic view of the second specimen; D: High-power microscopic view of the second specimen showing the structure of appendix with acute inflammatory infiltrate. Image credit: Abduallah Momtaz

Ultrasound revealed an inflamed appendix, causing cecal thickening with mild fluid around it and no other anomalies. An appendicitis diagnosis was made, and the patient was shifted to the operating room. During surgery, an incidental finding of a duplex appendix was made. The first appendix was an inflamed retro-cecal appendix adherent to the cecal wall entrapped with a peritoneal layer with cecum involving fecalith. The second appendix was an inflamed pre-ileal appendix. Appendectomy for both appendices was done (Figure 1). The clinical finding was confirmed by histopathological examination (Figure 2). The histopathological examination of the inflamed retro-cecal appendix revealed confluent mucosal erosions covered with fecalith and intense submucosal lymphoid aggregates. Acute inflammation with a predominance of neutrophils involves all layers of the appendiceal wall, with prominent edema, congestion, and focal hemorrhage. The peri-appendicular fat is markedly inflamed and congested, showing focal hemorrhage and multiple reactive peri-appendicular lymphoid follicles. This finding led to acute appendicitis with severe peri-appendicitis. Histopathological examination of the pre-ileal appendix demonstrated mucosal erosions covered with fecalith and blood with swelling and thickening of mucosa and submucosa by reactive lymphoid follicles. Peri-appendicular fat was congested. This finding led to acute appendicitis with lymphoid hyperplasia. The postoperative course and follow-ups after surgery were uneventful.

## Discussion

Appendiceal anomalies are among the rarest of their kind, with a double appendix having an incidence of 0.004% to 0.009%. They are usually detected unintentionally and intraoperatively the majority of the time [1-5].

**Figure 3:**
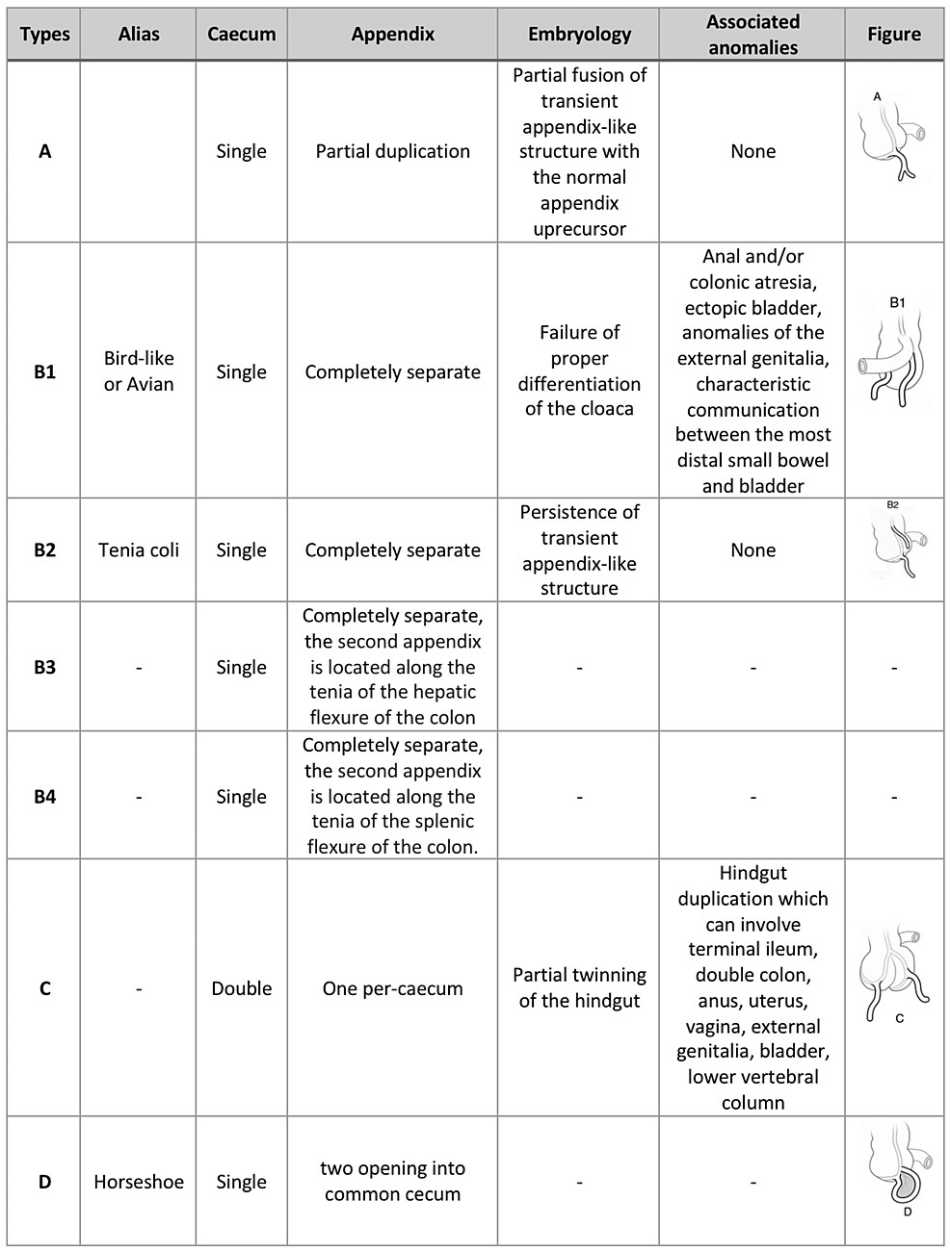
The Cave-Wallbridge classification Adapted from Raja and Vasanthakumaran [11].

Several classifications of appendiceal duplication have been established to classify these anomalies. Currently, the most widely accepted classification is the 'Cave-Wallbridge' classification, which was initially established by Cave in 1936 [7] and then refined by C. Waugh and Wallbridge in 1963 [8]. Biermann et al revised it again in 1993 [9]. The 'Cave-Wallbridge' classification classified appendicular anatomical variation into four broad categories based on appendicular location: A, B, C, and D (Figure 3). Category B is subdivided into B1, B2, B3, and B4 [2,10,11].

Type B2 is the most common and reported type, with no additional anomalies associated with it. Other types are associated with additional anomalies, such as duplications of the hindgut, colonic atresia, and bladder anomalies. This emphasizes the surgical importance of additional investigations to uncover potentially related anomalies [11]. Some highly unusual cases are discussed, such as the triple appendix [12], which cannot include the existing types.

Our case reported above applies under category B2 because there were two separate appendices seen to be arising from the caecum, one located retro-cecal and the other pre-ileal. The two were inflamed with no other anomaly around them. Since a double appendix is challenging to diagnose by standard imaging tests (ultrasonography and computed tomography) and cannot be assured by preoperative radiological examinations [2], our discovery of a double appendix was made intraoperatively. Our case was even more challenging since CT access was unavailable.

A double appendix might be asymptomatic or symptomatic due to obstruction or inflammation, even after an appendectomy to remove one of the two appendices or both. Associated abnormalities or duplications of the large intestine or the genitourinary system may be present in children, particularly in types B1 and C, which may act as "warning" indicators due to their similar embryological origin [2]. The recurrence of symptoms after appendectomy may also be due to stump appendicitis, a rare condition in which the residual appendix is inflamed [13].

We want to draw attention to the necessity of surgical staff being aware of appendix anomalies to avoid complications such as recurrent exploratory laparotomy if the second appendix becomes inflamed or ruptures. This results in generalized peritonitis. Furthermore, medical professionals need to understand the Cave-Wallbridge classification system when diagnosing appendiceal duplications, especially type B and C, as they may present additional anomalies that need further investigation [5].

## Conclusions

A right lower quadrant pain, fever, and leukocytosis are typically caused by appendicitis. If a patient has previously undergone an appendectomy, stump appendicitis and double appendices should be considered. In the case of a double appendix, both should be removed to prevent a later diagnostic dilemma. Despite the rarity of double appendices, a misdiagnosis can cause serious medical and legal complications.
